# Prognostic role of *PIK3CA* mutations and their association with hormone receptor expression in breast cancer: a meta-analysis

**DOI:** 10.1038/srep06255

**Published:** 2014-09-01

**Authors:** Bo Pang, Shi Cheng, Shi-Peng Sun, Cheng An, Zhi-Yuan Liu, Xue Feng, Gui-Jian Liu

**Affiliations:** 1 Clinical laboratory, Guang'anmen Hospital, China Academy of Chinese Medical Sciences, Beixian Ge 5#, XiCheng District, Beijing 100053, China

## Abstract

The phosphatidylinositol-4, 5-bisphosphate 3-kinase, catalytic subunit alpha (*PIK3CA*) gene is frequently mutated in breast cancer (BCa). Sex hormone receptors (HRs), including estrogen receptor (ER) and progesterone receptor (PR) play pivotal roles in BCa. In this study, we evaluated the association between *PIK3CA* mutations and ER/PR expression and the prognostic role of *PIK3CA* mutations in BCa patients, and in particular, HR-positive BCa. Thirty-two studies involving 5719 cases of BCa obtained from database searches were examined. *PIK3CA* gene mutations correlated significantly with ER/PR expression (*p* < 0.00001) and relapse-free survival (RFS) (hazard ratio [HR] 0.76, 95% confidence interval [CI] 0.59–0.98, *p* = 0.03) but not overall survival (OS) (HR 1.14, 95%CI 0.72–1.82, *p* = 0.57) in unsorted BCa patients. *PIK3CA* mutations were not associated with OS (HR 1.06, 95%CI 0.67–1.67, *p* = 0.81) or RFS (HR 0.86, 95%CI 0.53–1.40, *p* = 0.55) in HR-positive BCa patients. In conclusion, *PIK3CA* mutations were significantly related to ER/PR expression and RFS in unsorted BCa patients. However, the clinical implications of *PIK3CA* mutations may vary according to different mutant exons. And *PIK3CA* mutations alone may have limited prognostic value for HR-positive BCa patients.

Breast cancer (BCa) is one of the most common cancers among women, with more than 1,300,000 new cases and about 450,000 deaths reported each year worldwide^1^. This highly heterogeneous disease is divided into subgroups on the basis of molecular signatures, clinicopathologic features, and responses to therapy. Hormone receptors (HRs), including estrogen receptors (ERs) and progesterone receptors (PRs) are the most important markers of BCa. Most BCa cases are HR-positive (HR+), and ER-positive (ER+) BCa accounts for up to 80% of BCa cases among women 45 years and older^2^,^3^. Endocrine therapy is regarded as the cornerstone of ER+ BCa treatment. However, because of de novo or acquired resistance to endocrine therapy, prognosis is still poor for many ER+ BCa patients. Therefore, finding new effective treatment methods for ER+ BCa patients resistant to endocrine therapy is imperative.

After the *TP53* gene, the phosphatidylinositol-4,5-bisphosphate 3-kinase, catalytic subunit alpha (*PIK3CA*) gene is the most frequently mutated gene in BCa. Phosphatidylinositol 3-kinase (PI3K) is composed of an 85-kD (p85) and a 110-kD (p110) subunit. When coupled to activated tyrosine kinases via p85 (the adaptor subunit), p110 (the catalytic subunit) phosphorylates the 3-hydroxy group of inositol phospholipids. Gain-of-function mutations in *PIK3CA* have been found in different types of cancers including BCa. The mutations result in PI3K activation independent of upstream signaling and constitutive activation of the downstream AKT pathway and may contribute to oncogenesis^4^. The frequency of *PIK3CA* mutations in BCa cases ranges from 16.4 to 45%^5^. There are 3 mutation “hotspots” in the *PIK3CA* gene: E542K, E545K at exon 9 (helix domain) and H1047R at exon 20 (kinase domain). The 3 hotspots represent almost 80% of *PIK3CA* mutations and lead to constitutive PI3K activity by different mechanisms^6^.

Aberrant activation of the PI3K pathway is thought to contribute significantly to endocrine therapy resistance in patients with ER+ BCa^7^. There is evidence showing that endocrine therapy combined with p110 inhibitors is an effective treatment for ER+ BCa cases, including those with *PIK3CA* mutations^8^. The synthetic lethal interaction is a promising approach that needs further studies. Testing of several p110 inhibitors is underway in phase II clinical trials. Therefore, evaluation of the relationship between HRs and *PIK3CA* mutations in BCa is necessary. It is also of great clinical interest to determine whether *PIK3CA* mutations are prognostic factors in HR+ BCa patients.

## Results

### Search results and description of eligible studies

**Figure 1:**
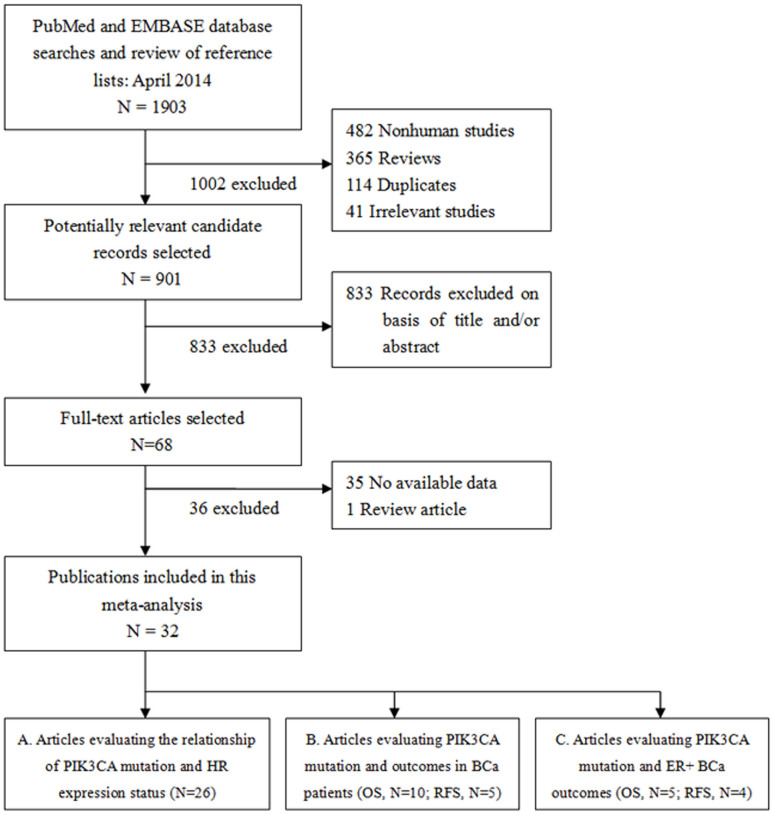
Summary flowchart of the literature search.

A total of 1903 potentially relevant citations were retrieved. After exclusion of nonhuman studies, reviews, and duplicates, two authors independently perused the titles and abstracts of the articles. After screenings, 68 articles were chosen for further full-text review. Ultimately, 32 eligible studies were included in our meta-analysis^5^,^9^,^10^,^11^,^12^,^13^,^14^,^15^,^16^,^17^,^18^,^19^,^20^,^21^,^22^,^23^,^24^,^25^,^26^,^27^,^28^,^29^,^30^,^31^,^32^,^33^,^34^,^35^,^36^,^37^,^38^,^39^ (Figure 1).

The 32 eligible studies were published from 2004 to 2014 and involved 5719 cases. Data from the studies were grouped as follows: group A evaluated the relationship between *PIK3CA* mutations and ER (26 studies) or PR (20 studies) expression in BCa patients, group B (12 studies) and group C (8 studies) evaluated the relationship between *PIK3CA* mutations and the outcomes of all BCa patients and HR+ BCa patients, respectively. In the 32 selected studies, the percentage of patients with *PIK3CA* mutations ranged from 7.1% to 44.6%, and the percentage of ER+ patients ranged from 48.1% to 84.0%. For PR, the percentage ranged from 41.4% to 64.8%. In the B and C groups, the median follow-up time ranged from 50 to 153.6 months.

### ER and PR expression and *PIK3CA* gene mutations in BCa patients

**Figure 2:**
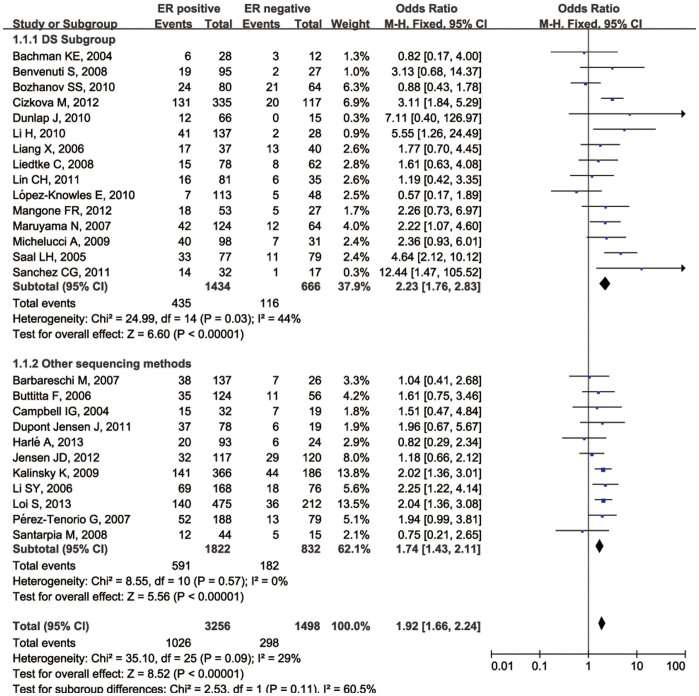
Forest plot with OR evaluating the relationship between *PIK3CA* mutation and ER expression status.

**Figure 3:**
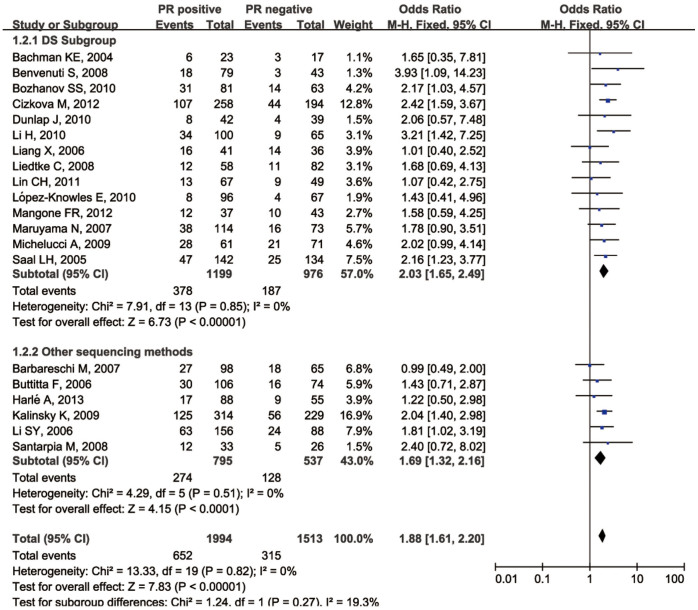
Forest plot with OR evaluating the relationship between *PIK3CA* mutation and PR expression status.

The relationship between *PIK3CA* gene mutations and ER expression was investigated in 4754 patients from 26 selected studies (Group A, the ER arm) using a fixed-effect model (Table 1). There was a significant association between *PIK3CA* gene mutations and ER expression in the patients in this group (odds ratio [OR] 1.92, 95%CI 1.65–2.23; *P* < 0.00001; Figure 2). Then we performed a separate analysis for PR expression in 3507 patients from 20 studies (Group A, the PR arm) using a fixed-effect model (Table 1), and found that PR expression was also significantly associated with *PIK3CA* mutations (OR 1.88, 95% CI 1.61–2.20; *P* < 0.00001) (Figure 3). Direct sequencing was the most frequently used method for detecting mutations in the selected studies. We introduced subgroups and found that direct sequencing and the other mutation detection methods produced similar results (*p* = 0.13).

### *PIK3CA* gene mutations and prognosis in all BCa patients

**Figure 4:**
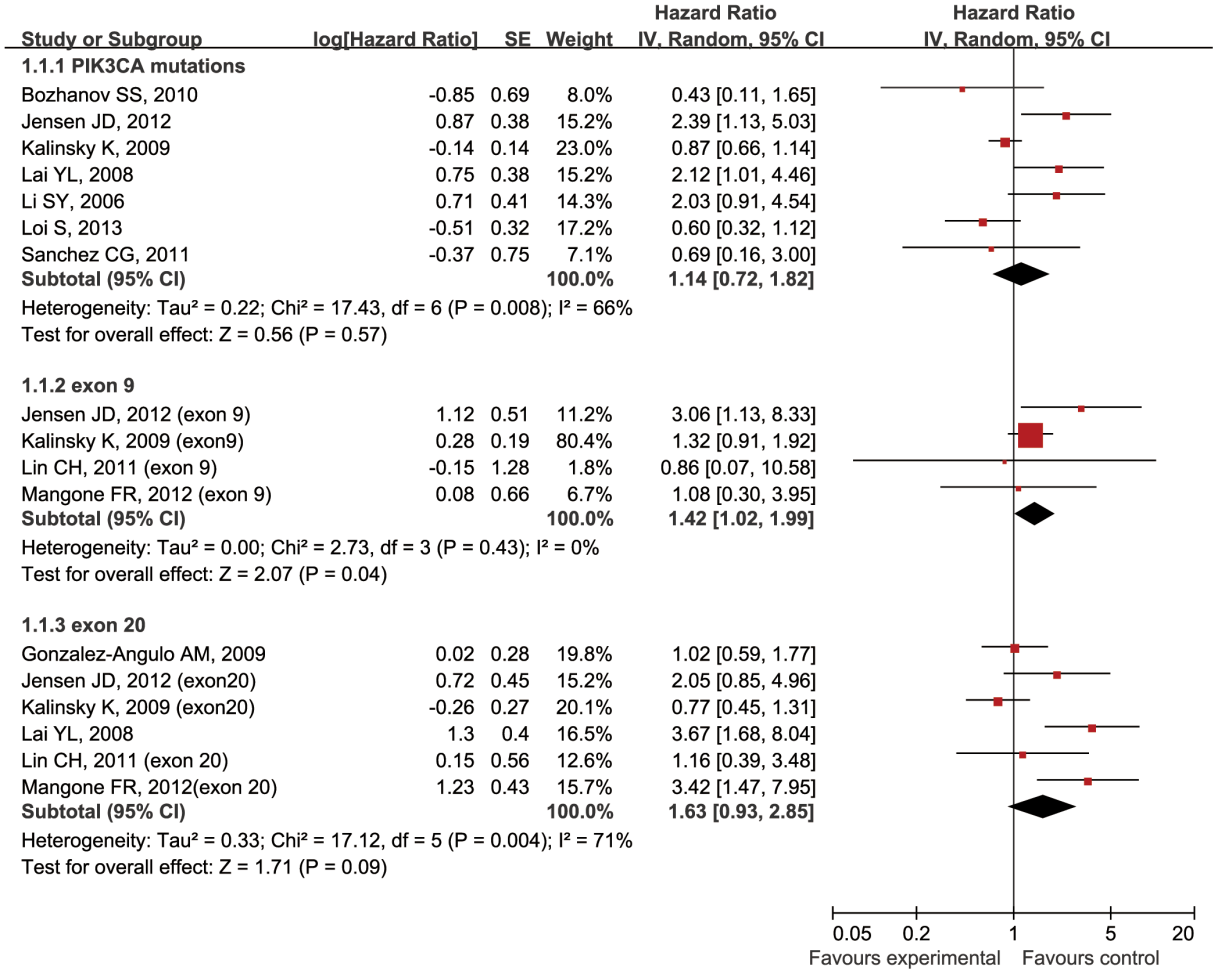
Forest plots of the analysis on the HR of OS in BCa patients. Subgroups are introduced for evaluating exon 9 or 20 mutations.

**Figure 5:**
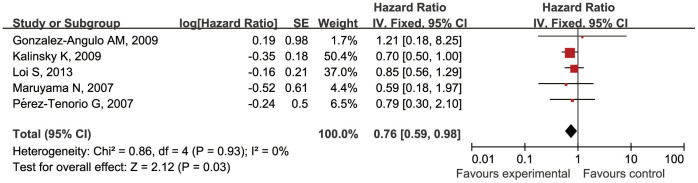
Forest plot of the analysis on the HR of RFS in BCa patients.

Analyses were conducted to evaluate the relationship between *PIK3CA* gene mutations and prognosis as defined by overall survival (OS) and relapse-free survival (RFS) in all BCa patients (group B) (Table 2). Because of significant heterogeneity among the group B studies for OS (*P* = 0.008; *I^2^* = 66%), a random-effect model was used to assess OS correlations. However, because there was no inter-study heterogeneity among the group B studies for RFS (*P* = 0.93; *I^2^* = 0%), a fixed-effect model was used to assess RFS correlations. For OS, 7 studies involving 2105 patients were analyzed and no significant association between *PIK3CA* mutations and OS was found (HR 1.14, 95% CI 0.72–1.82; *P* = 0.57) (Figure 4). We also performed analysis for different exons. For exon 9 mutations, a significant worse OS was found (HR 1.42, 95% CI 1.02–1.99; *P* = 0.04). In addition, for exon 20, the results of OS did not reach a significant level (HR 1.63, 95% CI 0.93–2.85; *P* = 0.09) (Figure 4). For RFS, 5 studies involving 1913 patients were analyzed, and a significant relationship between *PIK3CA* gene mutations and prolonged RFS was observed (hazard ratio 0.76, 95% CI 0.59–0.98; *P* = 0.03) (Fig. 5).

### *PIK3CA* gene mutations and prognosis in HR+ BCa patients

**Figure 6:**
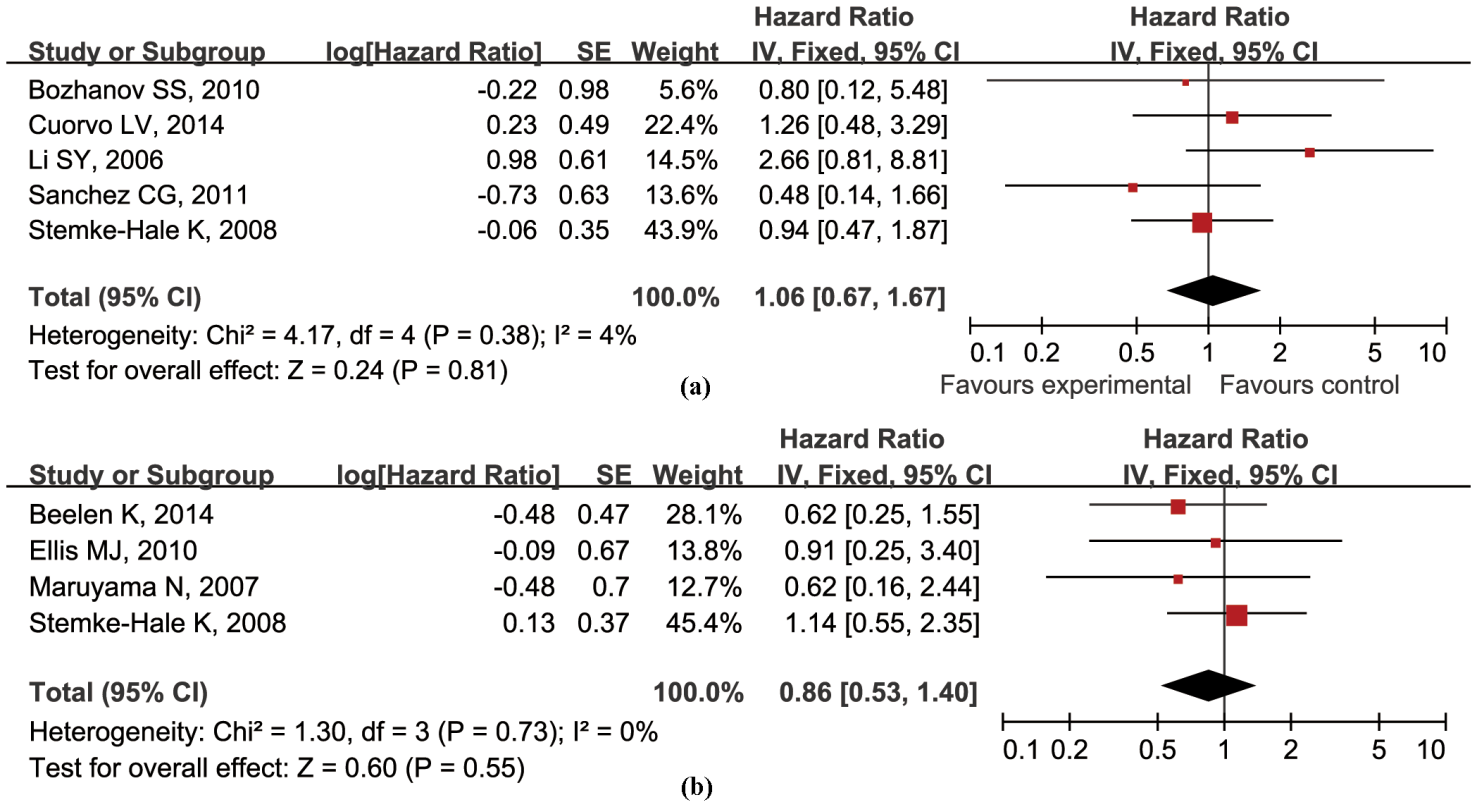
Forest plots of the analysis on the hazard ratio of OS (a) and RFS (b) in HR+ BCa patients.

The relationship between PIK3CA mutations and prognosis in HR+ BCa was evaluated in 8 studies involving 1021 patients, 5 studies (644 patients) for OS and 4 studies (534 patients) for RFS (group C) (Table 3). On the basis of the available data, kinase domain mutation is the priority for inclusion and analysis. No inter-study heterogeneity was found for OS (*P* = 0.38; *I^2^* = 4%) or RFS (*P* = 0.73; *I^2^* = 0%). *PIK3CA* gene mutations were not significantly associated with OS (hazard ratio 1.06, 95% CI 0.67–1.67; *P* = 0.81) (Fig. 6a) or RFS (hazard ratio 0.86, 95% CI 0.53–1.40; *P* = 0.55) (Fig. 6b) in HR+ BCa patients.

### Publication bias

Publication bias was not investigated when the number of studies was less than 10 because of the low sensitivity of qualitative and quantitative tests^40^. When the number of studies was more than 10, bias was assessed by Begg's funnel plots. No evidence of obvious asymmetry was found in this analysis by visual evaluation (data not shown).

## Discussion

Recently, several studies evaluating the prognosis of BCa patients suggest that *PIK3CA* mutations are “good mutations”. Our meta-analysis shows that *PIK3CA* gene mutations are significantly associated with both ER and PR expression, which are believed to be favorable clinicopathologic features of BCa. Furthermore, in unsorted BCa patients with *PIK3CA* mutations, RFS was significantly improved.

There are some possible explanations for the puzzling favorable effects of *PIK3CA* mutations. First, signaling pathways downstream of PI3K may not be active in some BCa patients with *PIK3CA* mutations. Loi et al. found that *PIK3CA* mutations were associated with relatively low mTORC1 signaling and that some AKT-regulated genes were repressed in BCa patients with *PIK3CA* mutations^31^. Second, dysregulated gene expression resulting from *PIK3CA* mutations may be advantageous. Cizkova showed that the Wnt pathway was dysregulated and WNT5A was overexpressed in ER+ BCa patients with *PIK3CA* mutations^41^. Interestingly, WNT5A expression has been associated with favorable outcomes in patients with invasive breast tumors^42^. Third, *PIK3CA*, like many other oncogenes, may induce senescence, resulting in a less aggressive phenotype after cell transformation^43^,^44^.

Despite of this, there was only an insignificant connection between *PIK3CA* mutations and OS. The improvement in RFS but not OS may suggest a BCa specific effect of *PIK3CA* mutations. However, considering specific exons, the effects seemed weak or even contradictory. In the future, more studies focusing on specific exons mutations, including the non-hotspot mutations of *PIK3CA*, are warranted.

Whether PIK3CA mutations contribute to endocrine therapy resistance remains unclear and intriguing. Another important finding of this study was that *PIK3CA* mutations did not affect either OS or RFS in HR+ BCa patients. In most of the studies selected for our analysis, hormone treatment was the standard therapy method. However, *PIK3CA* mutations may have only limited prognostic value with respect to hormone therapy responsiveness. Ellis et al. showed that the *PIK3CA* kinase domain mutations were inversely correlated with the clinical response to neoadjuvant endocrine treatment in BCa patients and was not associated with proliferation, as determined by immunostaining for Ki-67^20^. In patients who did not receive tamoxifen, as Beelen et al. showed, PIK3CA mutation was not a prognostic marker, either.

It also should be noted that there is some dissociation between *PIK3CA* mutations and activation of signaling pathways downstream of PI3K. In some phase I clinical trials, *PIK3CA* mutations were not strongly related to responses produced by PI3K inhibitors^17^. In our study, *PIK3CA* mutations were associated with favorable prognostic factors such as ER and PR expression, but are unlikely to be the single pivotal determinant of favorable responses to endocrine treatment. The gene signature associated with *PIK3CA* mutations was indicative of better clinical outcomes in ER+/HER2− BCa patients^45^. Perhaps its gene signature is more important than the *PIK3CA* mutation itself in respect to prognosis. Studies determining whether *PIK3CA* mutations are beneficial to tamoxifen-treated HR+ BCa patients with other molecular features such as PTEN loss or *AKT1* mutations are warranted.

There were some limitations to our study. First, we only analyzed available data in the literature. Second, because of significant heterogeneity, we used the random effect model, which is not as reliable as the fixed-effects model, in some analyses. Third, we only included articles that were published in English, and language bias might exist. Fourth, data extracted from the literature may not be as reliable as data generated directly. Fifth, several related studies of high quality were not included in our analysis because ideal unified prognosis parameters were lacking. Finally, the inclusion criteria and treatment procedures were not strictly unified in the studies used for our analysis. These differences are also a potential source of heterogeneity. Therefore, a cautious interpretation of our findings is warranted given possible bias in our meta-analysis.

In summary, our results show that *PIK3CA* mutations are significantly related to the ER and PR expression status of BCa patients. They also correlated with improved RFS in unsorted BCa patients, but not with OS or RFS in HR+ BCa patients. As a potential biomarker, *PIK3CA* mutations were not prognostic for HR+ BCa patients or, most notably, ER+ BCa patients. Future studies are needed that collectively explore the possible roles of *PIK3CA* mutations, the activation of signaling pathways downstream of PI3K, and other important biomarkers such as the genes encoding the components of the PI3K/AKT/mTOR pathway.

## Methods

### Literature search and eligibility criteria

We searched PubMed and Embase databases up to April 2014 for English-language titles or abstracts that included the words “phosphoinositide-3-kinase”, “*PIK3CA*”, “mutation”, “breast cancer”, or “breast neoplasms”. We also screened the references of the retrieved articles and relevant reviews for additional articles. A published article was included if it (1) evaluated the association between *PIK3CA* mutations and ER or PR expression in BCa patients or the association between *PIK3CA* mutations and BCa prognosis; (2) had sufficient data for estimating an OR with a 95% CI or a HR with a 95% CI; and (3) evaluated OS, RFS, or other survival index. The exclusion criteria were as follows: (1) letters, reviews, conference abstracts, and case reports; and (2) articles that did not provide sufficient information such as a HR for OS or had data that could not be extracted.

### Data extraction and quality assessment

Two authors independently screened all publications by title or abstract for inclusion in our study. Discrepancies were resolved by group discussion, and data were extracted from eligible publications. The following information was collected: name of the first author, year of publication, source of patients, study design, mean age of the patients, percentage of ER+ and PR+ patients, percentage of patients with *PIK3CA* mutations, the region of the sequenced *PIK3CA* mutations, mutation analysis methods, outcome of BCa patients, and median follow-up time (months, range). The studies were assessed for quality according to the Newcastle-Ottawa quality assessment scale, and articles with 5 stars or more qualified for our study^46^.

### Statistical analysis

An OR with a 95% CI was used to assess the strength of the association between *PIK3CA* mutations and ER or PR expression status. The primary end points were RFS and OS. A HR and a 95% CI were used to estimate the impact of *PIK3CA* mutations on RFS and OS. When a HR and a 95% CI were not given in the article, estimated values were derived indirectly from Kaplan-Meier curves using the methods described by Tierney et al.^47^. Kaplan-Meier curves were read by an Engauge Digitizer, version 4.1 (http://digitizer.sourceforge.net/), and the data from the curves were entered in the spreadsheet appended to Tierney's report^47^. A combined HR > 1 implied a worse survival for groups of patients with *PIK3CA* mutations. Cochran Q and *I^2^* statistic values were used to assess heterogeneity among the studies. For the Q statistic, a *P* value < 0.10 was considered statistically significant for heterogeneity^48^, and the random effects model was calculated according to the DerSimonian-Laird method^49^.Otherwise, the fixed-effects model (Mantel-Haenszel method) was used. *I^2^* < 50% was considered acceptable. If significant heterogeneity was found, a random-effects model was used for meta-analysis. Statistical analyses were performed using Review Manager 5.0 software (http://www.cochrane.org). A significant two-way *P* value for comparison was defined as *P* < 0.05.

### Ethical Standards

We declare that the experiments comply with the current laws of China.

## Author Contributions

B.P. carried out the search of the Embase and Pubmed database, performed the statistical analysis by Revman, participated in the design of the study and drafted the manuscript. S.C. carried out the search of the Embase and Pubmed database and performed the statistical analysis by Revman. S.P.S. performed the data collection and extraction and helped to draft the manuscript. C.A. participated in the design of the study and made the language polishing. Z.Y.L. performed the data collection, extraction and arrangement. X.F. performed the data collection and arrangement. G.J.L. conceived of the study, and participated in its design and coordination and helped to draft the manuscript.

## Figures and Tables

**Table 1:** Main characteristics of studies that evaluated the relationship of *PIK3CA* mutations and ER/PR status in breast cancer patients

First author	Year of publication	Country	Design	Mean age(years)	No.of ER positive patients (%)	No.of PR positive patients (%)	No.of *PIK3CA* mutant patients (%)	Sequenced *PIK3CA*	Mutation analysis methods
Bachman KE	2004	USA	HB	NR	28 (68.3)	23(57.5)	9 (22.0)	exon 1,9 and 20	DS
Benvenuti S	2008	Italy	HB	NR	95 (76.0)	79(64.8)	28(16.0)	exon 9 and 20	DS
Bozhanov SS	2010	Bulgaria	HB	NR	81 (55.9)	81(56.3)	45 (31.3)	exon 9 and 20	DS
Cizkova M	2012	France	HB	61.6 (31–91)	335 (74.1)	258(57.1)	151 (33.4)	exon 9 and 20	DS
Dunlap J	2010	USA	HB	NR	66 (81.5)	42(51.9)	12 (14.8)	exon 7,9 and 20	DS
Li H	2010	China	HB	51 (33–80)	137 (83.0)	100(60.6)	43 (26.1)	exon 9 and 20	DS
Liang X	2006	Singapore	HB	NR	37 (48.1)	41(53.2)	31 (38.8)	exon 9 and 20	DS
Liedtke C	2008	USA	HB (stage II–III)	51 (28–73)	78 (55.7)	58(41.4)	23 (16.4)	exon 1,9 and 20	DS
Lin CH	2011	China(Taiwan)	HB	NR (less than 35 y)	81 (69.8)	67(57.8)	22 (19.0)	exon 9 and 20	DS
López-Knowles E	2010	Australia	HB	54*	113 (70.2)	96(58.9)	12 (7.1)	exon 9 and 20	DS
Mangone FR	2012	Brazil	HB	55 (26–85)	53 (61.6)	37(46.3)	22 (30.6)	exon 9 and 20	DS
Maruyama N	2007	Japan	HB	NR	124 (66.0)	114(61.0)	54(28.7)	exon 1, 2, 4, 7, 9, 13, 18, and 20	DS
Michelucci A	2009	Italy	HB	43.5 (32–61)	98 (76.0)	88(61.5)	63 (35.8)	exon 9 and 20	DS
Saal LH	2005	USA	HB	59 (24–89)	162 (55.5)	142(51.4)	77 (26.4)	exon 1, 2, 4, 5, 7, 9,12,13,18, 20	DS
Sanchez CG	2011	USA	HB	53.4 (32–80)	32 (62.7)	NR	16 (31.4)	exon 9 and 20 (HS)	DS
Barbareschi M	2007	Italy	HB	62 (17–89)	137 (84.0)	98(60.1)	45 (27.6)	exon 9 and 20	SSCP + DS
Buttitta F	2006	Italy	HB	57.2*	124 (68.9)	106(58.9)	46 (25.6)	exon 1–20	SSCP + DS
Campbell IG	2004	Australia	HB	NR	32 (62.7)	NR	22(43.1)	exon 1–20	SSCP + DHPLC
Dupont Jensen J	2011	Denmark	HB	57 (32–87)	78 (77.2)	NR	45 (44.6)	exon 9 and 20 (HS)	SNaPshot/DxS
Harlé A	2013	France	HB	NR	113 (79.0)	88(61.5)	26(18.2)	exon 9 and 20 (HS)	PCR-ARMS
Jensen JD	2012	Denmark	HB (HER2+)	NR	118 (49.4)	NR	61 (25.7)	exon 9 and 20	PA
Kalinsky K	2009	USA	HB	NR	366 (62.0)	314(57.8)	192 (32.5)	exon 1–20	SM + SS
Li SY	2006	Australia	HB	59 (18–93)	168 (68.9)	156(63.9)	88 (35.2)	exon 7,9 and 20	F-SSCP
Loi S	2013	Finnish	HB	NR	475 (69.1)	NR	174 (25.3)	exons 1, 2, 4, 9, 13, 18, 20	SM
Pérez-Tenorio G	2007	Sweden	HB	NR	188 (70.4)	NR	65(24.3)	exon 9 and 20	SSCP + DS
Santarpia M	2008	Italy/Spain	HB	58 (32–85)	44 (74.6)	33(55.9)	17 (27.9)	exon 9 and 20 (HS)	AD

NR, not reported; HB, hospital based group; HS, hotspots mutation; AD, allelic discrimination; DHPLC, denaturing high performance liquid chromatography; DS, direct sequencing; SNaPshot, SNaPshot genotyping assay; DxS, DxS PI3K mutation test kit; F-SSCP, Fluorescent Single-Strand Conformation Polymorphism; PA, pyrosequencing assay; PCR-Amplification Refractory Mutation System (PCR-ARMS); SM, Sequenom MassARRAY; SS,Sanger sequencing.

*means that the ranges of age were not reported in the studies.

**Table 2:** Main characteristics of studies that evaluated the relationships of *PIK3CA* mutations and the OS/RFS in breast cancer patients

First author	Year of publication	Country	Design	Treatment	No.of *PIK3CA* mutant patients (%)	Sequenced *PIK3CA*	Mutation analysis methods	Median follow-up time (months, range)	Outcomes
Bozhanov SS	2010	Bulgaria	HB	H, C, RT	45 (31.3)	exon 9 and 20	DS	69 (11–96)	OS
Jensen JD	2012	Denmark	HB(Her2+)	H, C, T	61 (25.7)	exon 9 and 20	PA	67*	OS
Kalinsky K	2009	USA	HB	NR	192 (32.5)	exon 1–20	SM + SS	153.6*	OS, RFS
Lai YL	2008	China (Taiwan)	HB	H, C, RT	39 (25.7)	exon 4, 7, 9 and 20	DS	78 (1.3–113.2)	OS
Li SY	2006	Australia	HB	H, C	88 (35.2)	exon 7,9 and 20	F-SSCP	50 (2–78)	OS
Loi S	2013	Finnish	HB	H, C, T	174 (25.3)	exons 1, 2, 4, 9, 13, 18, 20	SM	62*	OS, RFS
Sanchez CG	2011	USA	HB	NR	16 (31.4)	exon 9 and 20 (HS)	DS	51.7 (0.9–256.7)	OS
Lin CH	2011	China (Taiwan)	HB	H, C	22 (19.0)	exon 9 and 20	DS	62.7*	OS
Mangone FR	2012	Brazil	HB	NR	22 (30.6)	exon 9 and 20	DS	63.3 (25–78)	OS
Gonzalez-Angulo AM	2009	USA	HB	H, C	78 (22.5)	exon 9 and 20	SM	50.4 (9.6–110.4)	OS, RFS
Maruyama N	2007	Japan	HB	H, C	54 (28.7)	exon 1, 2, 4, 7, 9, 13, 18, 20	DS	64 (38–88)	RFS
Pérez-Tenorio G	2007	Sweden	HB	H, C, RT	65 (24.1)	exon 9 and 20	SSCP + DS	132*	RFS

*means that the ranges of age or months were not reported in the studies.

C, Chemotherapy; T: Trastuzumab; H, Hormonal therapy; RT, Radiothrapy.

**Table 3:** Main characteristics of studies that evaluated the relationships of *PIK3CA* mutations and the OS/RFS in HR+ breast cancer patients

First author	Year of publication	Country	Design	Treatment	No.of *PIK3CA* mutant patients (%)	Sequenced *PIK3CA*	Mutation analysis methods	Median follow-up time (months, range)	Outcome type
Bozhanov SS	2010	Bulgaria	HB	H, C, RT	24(30.0)	exon 9 and 20	DS	69 (11–96)	OS
Cuorvo LV	2014	Italy	HB	H, C, T	50(20.3)	exon 9 and 20	HRM + PA	97 (8–140)	OS*
Li SY	2006	Australia	HB	H, C	69(41.1)	exon 7, 9 and 20	F-SSCP	50 (2–78)	OS
Sanchez CG	2011	USA	HB	NR	13(48.1)	exon 9 and 20 (HS)	DS	51.7 (0.9–256.7)	OS
Stemke-Hale K	2008	Spain, Netherlands and USA	HB	H	80(34.5)	23 known mutations	MS	NR	OS, RFS
Beelen K	2014	Netherlands	HB	Control arm	28(25.2)	exon 9 and 20 (HS)	SM	93.6	RFS
Ellis MJ	2010	Multicentre	HB	H	45(29.4)	exon 9 and 20	DS	NR	RFS*
Maruyama N	2007	Japanese	HB	H, C	54(28.7)	exon 1, 2, 4, 7, 9, 13, 18, and 20	DS	64 (38–88)	RFS

C, Chemotherapy; T: Trastuzumab; H, Hormonal therapy; RT, Radiothrapy; HRM, high resolution melting analysis.

*only exon 20 mutations were analyzed.

## References

[b1] Network C. G. A. Comprehensive molecular portraits of human breast tumours. Nature 490, 61–70 (2012). 23000897 10.1038/nature11412 PMC3465532

[b2] Boswell K. A., Wang X., Shah M. V. & Aapro M. S. Disease burden and treatment outcomes in second-line therapy of patients with estrogen receptor-positive (ER+) advanced breast cancer: a review of the literature. Breast 21, 701–706 (2012). 23092824 10.1016/j.breast.2012.09.005

[b3] Glass A. G., Lacey J. V. Jr, Carreon J. D. & Hoover R. N. Breast cancer incidence, 1980-2006: combined roles of menopausal hormone therapy, screening mammography, and estrogen receptor status. J Natl Cancer Inst 99, 1152–1161 (2007). 17652280 10.1093/jnci/djm059

[b4] O'Brien C. *et al.* Predictive biomarkers of sensitivity to the phosphatidylinositol 3′ kinase inhibitor GDC-0941 in breast cancer preclinical models. Clin Cancer Res 16, 3670–3683 (2010). 20453058 10.1158/1078-0432.CCR-09-2828

[b5] Mangone F. R., Bobrovnitchaia I. G., Salaorni S., Manuli E. & Nagai M. A. PIK3CA exon 20 mutations are associated with poor prognosis in breast cancer patients. Clinics (Sao Paulo) 67, 1285–1290 (2012). 23184205 10.6061/clinics/2012(11)11 PMC3488987

[b6] Drury S. C. *et al.* Changes in breast cancer biomarkers in the IGF1R/PI3K pathway in recurrent breast cancer after tamoxifen treatment. Endocr Relat Cancer 18, 565–577 (2011). 21734071 10.1530/ERC-10-0046

[b7] Boulay A. *et al.* Dual inhibition of mTOR and estrogen receptor signaling in vitro induces cell death in models of breast cancer. Clin Cancer Res 11, 5319–5328 (2005). 16033851 10.1158/1078-0432.CCR-04-2402

[b8] Crowder R. J. *et al.* PIK3CA and PIK3CB inhibition produce synthetic lethality when combined with estrogen deprivation in estrogen receptor-positive breast cancer. Cancer Res 69, 3955–3962 (2009). 19366795 10.1158/0008-5472.CAN-08-4450 PMC2811393

[b9] Bachman K. E. *et al.* The PIK3CA gene is mutated with high frequency in human breast cancers. Cancer Biol Ther 3, 772–775 (2004). 15254419 10.4161/cbt.3.8.994

[b10] Barbareschi M. *et al.* Different prognostic roles of mutations in the helical and kinase domains of the PIK3CA gene in breast carcinomas. Clin Cancer Res 13, 6064–6069 (2007). 17947469 10.1158/1078-0432.CCR-07-0266

[b11] Beelen K. *et al.* PIK3CA mutations, phosphatase and tensin homolog, human epidermal growth factor receptor 2 and insulin-like growth factor 1 receptor and adjuvant tamoxifen resistance in postmenopausal breast cancer patients. Breast Cancer Res 16, R13 (2014). 24467828 10.1186/bcr3606 PMC3978618

[b12] Benvenuti S. *et al.* PIK3CA cancer mutations display gender and tissue specificity patterns. Hum Mutat 29, 284–288 (2008). 18022911 10.1002/humu.20648

[b13] Bozhanov S. S. *et al.* Alterations in p53, BRCA1, ATM, PIK3CA, and HER2 genes and their effect in modifying clinicopathological characteristics and overall survival of Bulgarian patients with breast cancer. J Cancer Res Clin Oncol 136, 1657–1669 (2010). 20177704 10.1007/s00432-010-0824-9 PMC11828217

[b14] Buttitta F. *et al.* PIK3CA mutation and histological type in breast carcinoma: high frequency of mutations in lobular carcinoma. J Pathol 208, 350–355 (2006). 16353168 10.1002/path.1908

[b15] Campbell I. G. *et al.* Mutation of the PIK3CA gene in ovarian and breast cancer. Cancer Res 64, 7678–7681 (2004). 15520168 10.1158/0008-5472.CAN-04-2933

[b16] Cizkova M. *et al.* PIK3CA mutation impact on survival in breast cancer patients and in ERalpha, PR and ERBB2-based subgroups. Breast Cancer Res 14, R28 (2012). 22330809 10.1186/bcr3113 PMC3496146

[b17] Cuorvo L. V. *et al.* PI3KCA mutation status is of limited prognostic relevance in ER-positive breast cancer patients treated with hormone therapy. Virchows Archiv: an international journal of pathology 464, 85–93 (2014). 24233241 10.1007/s00428-013-1500-7

[b18] Dunlap J. *et al.* Phosphatidylinositol-3-kinase and AKT1 mutations occur early in breast carcinoma. Breast Cancer Res Treat 120, 409–418 (2010). 19418217 10.1007/s10549-009-0406-1

[b19] Dupont Jensen J. *et al.* PIK3CA mutations may be discordant between primary and corresponding metastatic disease in breast cancer. Clin Cancer Res 17, 667–677 (2011). 20940279 10.1158/1078-0432.CCR-10-1133

[b20] Ellis M. J. *et al.* Phosphatidyl-inositol-3-kinase alpha catalytic subunit mutation and response to neoadjuvant endocrine therapy for estrogen receptor positive breast cancer. Breast Cancer Res Treat 119, 379–390 (2010). 19844788 10.1007/s10549-009-0575-y PMC2810126

[b21] Gonzalez-Angulo A. M. *et al.* Androgen receptor levels and association with PIK3CA mutations and prognosis in breast cancer. Clin Cancer Res 15, 2472–2478 (2009). 19276248 10.1158/1078-0432.CCR-08-1763

[b22] Harle A. *et al.* Analysis of PIK3CA exon 9 and 20 mutations in breast cancers using PCR-HRM and PCR-ARMS: correlation with clinicopathological criteria. Oncology reports 29, 1043–1052 (2013). 23314198 10.3892/or.2013.2229

[b23] Jensen J. D. *et al.* PIK3CA mutations, PTEN, and pHER2 expression and impact on outcome in HER2-positive early-stage breast cancer patients treated with adjuvant chemotherapy and trastuzumab. Ann Oncol 23, 2034–2042 (2012). 22172323 10.1093/annonc/mdr546

[b24] Kalinsky K. *et al.* PIK3CA mutation associates with improved outcome in breast cancer. Clin Cancer Res 15, 5049–5059 (2009). 19671852 10.1158/1078-0432.CCR-09-0632

[b25] Lai Y. L. *et al.* PIK3CA exon 20 mutation is independently associated with a poor prognosis in breast cancer patients. Ann Surg Oncol 15, 1064–1069 (2008). 18183466 10.1245/s10434-007-9751-7

[b26] Li H. *et al.* PIK3CA mutations mostly begin to develop in ductal carcinoma of the breast. Exp Mol Pathol 88, 150–155 (2010). 19818761 10.1016/j.yexmp.2009.09.016

[b27] Li S. Y., Rong M., Grieu F. & Iacopetta B. PIK3CA mutations in breast cancer are associated with poor outcome. Breast Cancer Res Treat 96, 91–95 (2006). 16317585 10.1007/s10549-005-9048-0

[b28] Liang X. *et al.* Mutational hotspot in exon 20 of PIK3CA in breast cancer among Singapore Chinese. Cancer Biol Ther 5, 544–548 (2006). 16582596 10.4161/cbt.5.5.2656

[b29] Liedtke C. *et al.* PIK3CA-activating mutations and chemotherapy sensitivity in stage II–III breast cancer. Breast Cancer Res 10, R27 (2008). 18371219 10.1186/bcr1984 PMC2397526

[b30] Lin C. H. *et al.* Prognostic molecular markers in women aged 35 years or younger with breast cancer: is there a difference from the older patients? J Clin Pathol 64, 781–787 (2011). 21642658 10.1136/jclinpath-2011-200064

[b31] Loi S. *et al.* Somatic mutation profiling and associations with prognosis and trastuzumab benefit in early breast cancer. J Natl Cancer Inst 105, 960–967 (2013). 23739063 10.1093/jnci/djt121 PMC3699437

[b32] Lopez-Knowles E. *et al.* PI3K pathway activation in breast cancer is associated with the basal-like phenotype and cancer-specific mortality. Int J Cancer 126, 1121–1131 (2010). 19685490 10.1002/ijc.24831

[b33] Maruyama N. *et al.* Clinicopathologic analysis of breast cancers with PIK3CA mutations in Japanese women. Clin Cancer Res 13, 408–414 (2007). 17202311 10.1158/1078-0432.CCR-06-0267

[b34] Michelucci A. *et al.* PIK3CA in breast carcinoma: a mutational analysis of sporadic and hereditary cases. Diagn Mol Pathol 18, 200–205 (2009). 19861897 10.1097/PDM.0b013e31818e5fa4

[b35] Perez-Tenorio G. *et al.* PIK3CA mutations and PTEN loss correlate with similar prognostic factors and are not mutually exclusive in breast cancer. Clin Cancer Res 13, 3577–3584 (2007). 17575221 10.1158/1078-0432.CCR-06-1609

[b36] Saal L. H. *et al.* PIK3CA mutations correlate with hormone receptors, node metastasis, and ERBB2, and are mutually exclusive with PTEN loss in human breast carcinoma. Cancer Res 65, 2554–2559 (2005). 15805248 10.1158/0008-5472-CAN-04-3913

[b37] Sanchez C. G. *et al.* Preclinical modeling of combined phosphatidylinositol-3-kinase inhibition with endocrine therapy for estrogen receptor-positive breast cancer. Breast Cancer Res 13, R21 (2011). 21362200 10.1186/bcr2833 PMC3219179

[b38] Santarpia M. *et al.* PIK3CA mutations and BRCA1 expression in breast cancer: potential biomarkers for chemoresistance. Cancer Invest 26, 1044–1051 (2008). 18798071 10.1080/07357900802112701

[b39] Stemke-Hale K. *et al.* An integrative genomic and proteomic analysis of PIK3CA, PTEN, and AKT mutations in breast cancer. Cancer Res 68, 6084–6091 (2008). 18676830 10.1158/0008-5472.CAN-07-6854 PMC2680495

[b40] Ioannidis J. P. & Trikalinos T. A. The appropriateness of asymmetry tests for publication bias in meta-analyses: a large survey. CMAJ 176, 1091–1096 (2007). 17420491 10.1503/cmaj.060410 PMC1839799

[b41] Cizkova M. *et al.* Gene expression profiling reveals new aspects of PIK3CA mutation in ERalpha-positive breast cancer: major implication of the Wnt signaling pathway. PLoS One 5, e15647 (2010). 21209903 10.1371/journal.pone.0015647 PMC3012715

[b42] Ford C. E., Ekstrom E. J., Howlin J. & Andersson T. The WNT-5a derived peptide, Foxy-5, possesses dual properties that impair progression of ERalpha negative breast cancer. Cell Cycle 8, 1838–1842 (2009). 19448401 10.4161/cc.8863

[b43] Campisi J. & d'Adda di Fagagna F. Cellular senescence: when bad things happen to good cells. Nat Rev Mol Cell Biol 8, 729–740 (2007). 17667954 10.1038/nrm2233

[b44] Dumont A. G., Dumont S. N. & Trent J. C. The favorable impact of PIK3CA mutations on survival: an analysis of 2587 patients with breast cancer. Chin J Cancer 31, 327–334 (2012). 22640628 10.5732/cjc.012.10032 PMC3777497

[b45] Loi S. *et al.* PIK3CA mutations associated with gene signature of low mTORC1 signaling and better outcomes in estrogen receptor-positive breast cancer. Proc Natl Acad Sci U S A 107, 10208–10213 (2010). 20479250 10.1073/pnas.0907011107 PMC2890442

[b46] Stang A. Critical evaluation of the Newcastle-Ottawa scale for the assessment of the quality of nonrandomized studies in meta-analyses. Eur J Epidemiol 25, 603–605 (2010). 20652370 10.1007/s10654-010-9491-z

[b47] Tierney J. F., Stewart L. A., Ghersi D., Burdett S. & Sydes M. R. Practical methods for incorporating summary time-to-event data into meta-analysis. Trials 8, 16 (2007). 17555582 10.1186/1745-6215-8-16 PMC1920534

[b48] Lau J., Ioannidis J. P. & Schmid C. H. Quantitative synthesis in systematic reviews. Ann Intern Med 127, 820–826 (1997). 9382404 10.7326/0003-4819-127-9-199711010-00008

[b49] DerSimonian R. & Laird N. Meta-analysis in clinical trials. Control Clin Trials 7, 177–188 (1986). 3802833 10.1016/0197-2456(86)90046-2

